# Escherichia coli Pyogenic Ventriculitis in an Infant Following Abdominal Surgery: A Rare Case Report

**DOI:** 10.7759/cureus.84558

**Published:** 2025-05-21

**Authors:** Mohammed Maarad, Marwane Alaraibi, Mohamed Dahamou, Houssam Bkiyar, Brahim Housni

**Affiliations:** 1 Intensive Care, Mohammed First University, Oujda, Oujda, MAR; 2 Neurosurgery, Mohammed First University, Oujda, Oujda, MAR

**Keywords:** escherichia coli, infant, mri diagnosis, postoperative infection, pyogenic ventriculitis

## Abstract

Pyogenic ventriculitis is a severe and uncommon bacterial infection of the brain's ventricular system, most often associated with neurosurgical procedures or trauma, but it can rarely occur in other clinical settings. This case report describes an unusual presentation in an eight-month-old infant with no prior medical history, who developed this condition following abdominal surgery for acute intussusception. After an initially uneventful postoperative course, the patient presented with persistent high fever, seizures, and neurological deterioration. Diagnostic imaging revealed triventricular hydrocephalus on computed tomography (CT), and magnetic resonance imaging (MRI) confirmed the presence of pyogenic ventriculitis. Laboratory analysis of cerebrospinal fluid and blood cultures identified multidrug-resistant *Escherichia coli* as the pathogen. The infant was treated with mechanical ventilation, Anti-seizure medications, corticosteroids, broad-spectrum antibiotics, and external ventricular drainage. Clinical improvement was observed, and the patient was transferred for continued care. This case highlights the importance of considering central nervous system infections in infants with atypical postoperative courses and reinforces the value of MRI in early and accurate diagnosis, as well as the need for prompt, multidisciplinary management.

## Introduction

Pyogenic ventriculitis is a severe infection of the brain's ventricular system, typically associated with neurosurgical procedures or head trauma, though rare cases have also been reported in the general population [1]. In infants, ventriculitis is most often observed in the context of neurosurgical interventions or traumatic brain injury. However, cases arising from non-neurosurgical sources, such as abdominal surgery and postoperative sepsis, are exceedingly rare. This case is unique due to the development of pyogenic ventriculitis secondary to abdominal surgery in an otherwise healthy infant, underscoring the importance of considering this diagnosis in atypical clinical scenarios.

Early diagnosis and prompt identification of the causative bacteria are essential to improving clinical outcomes, with magnetic resonance imaging (MRI) with contrast widely regarded as the most reliable diagnostic modality [1]. While cerebrospinal fluid (CSF) and blood cultures remain the primary tools for identifying the responsible pathogen, they can occasionally yield false-negative results, thereby delaying the initiation of appropriate therapy [2].

Here, we report the case of an 8-month-old infant with no prior medical history who was admitted for acute intussusception and subsequently developed pyogenic ventriculitis caused by *Escherichia coli*, secondary to postoperative sepsis.

## Case presentation

An eight-month-old male infant with no notable medical history was admitted to the pediatric emergency department due to persistent paroxysmal irritability accompanied by non-bilious vomiting. On presentation, the infant was conscious, with stable hemodynamic and respiratory parameters, and the clinical assessment was further marked by the presence of blood-stained stools, which prompted abdominal ultrasonography that confirmed the diagnosis of acute intussusception.

The patient underwent surgical reduction of the affected bowel loop following failed air enema reduction, during which intestinal viability was verified and a Redon drain was placed. The immediate postoperative course was uneventful, marked by a favorable clinical evolution, resumption of oral feeding and bowel function within two days, and subsequent removal of the drain.

**Figure 1:**
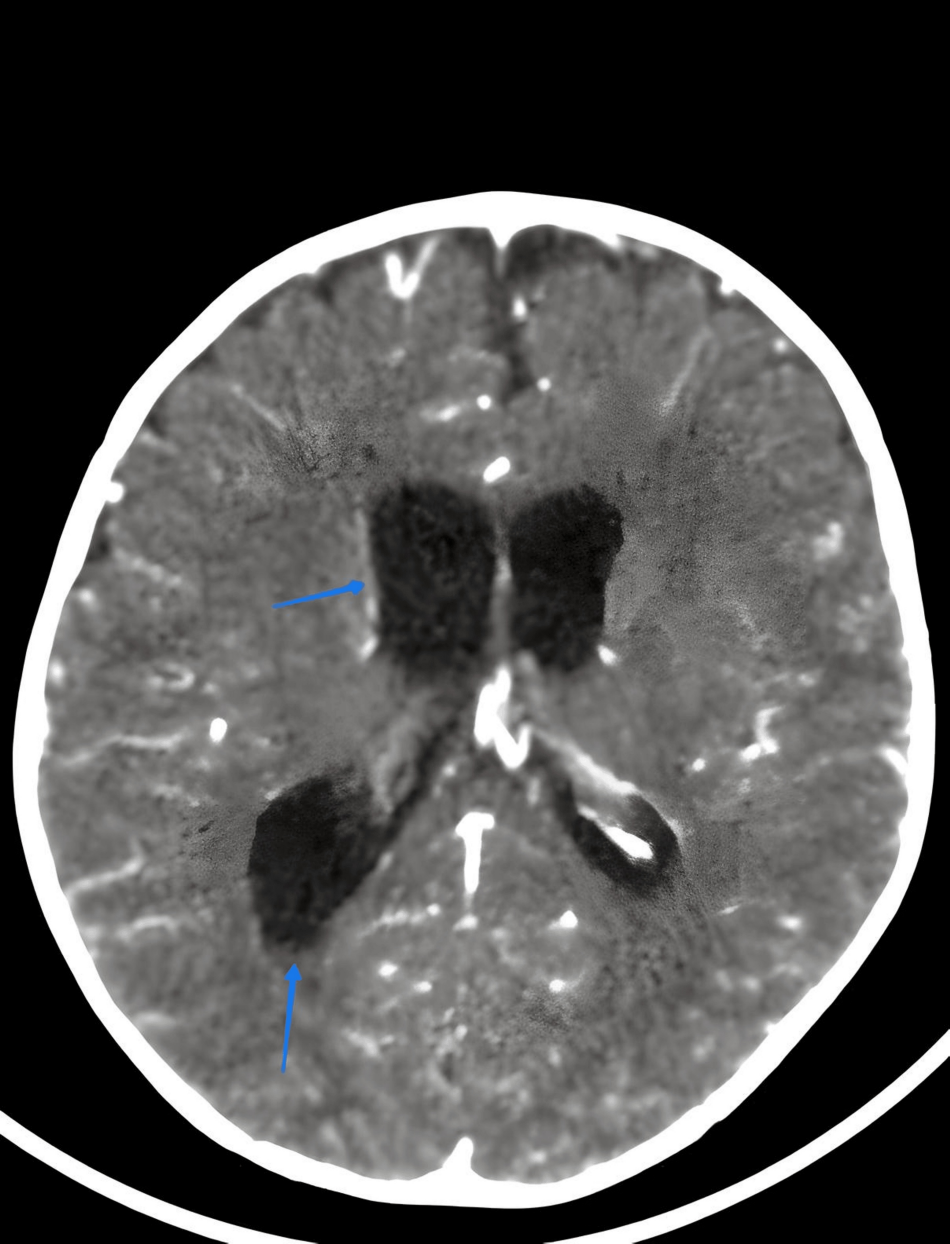
Axial contrast-enhanced brain CT showing triventricular hydrocephalus with right ventricular wall enhancement and occipital horn sediment suggestive of ventriculitis.

**Figure 2:**
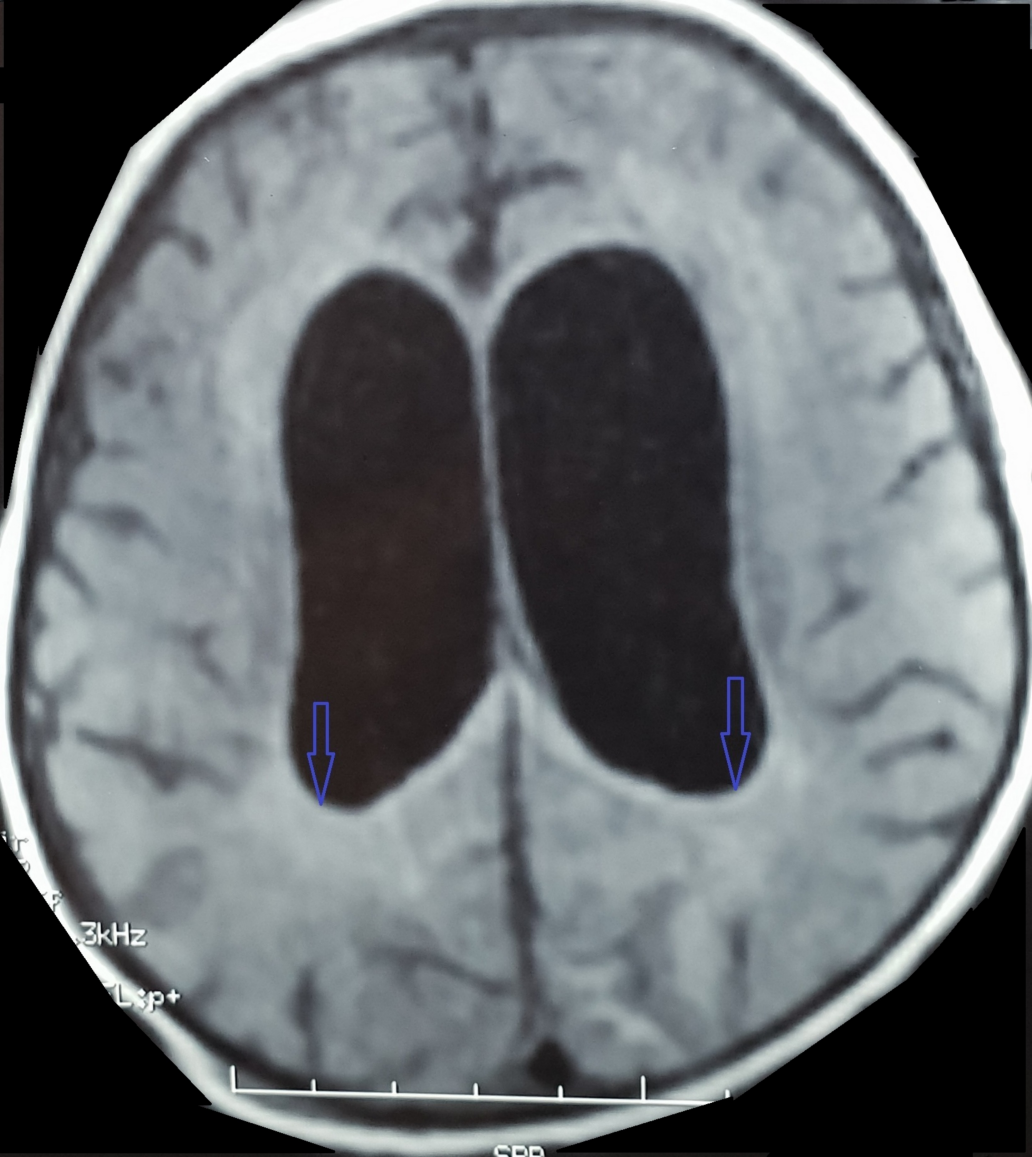
Axial cerebral MRI with FLAIR sequence showing ventriculitis.

During the first postoperative week, the infant developed recurrent febrile episodes, with temperatures reaching up to 40°C. There were no signs of peritonitis, wound infection before the onset of central nervous system (CNS) symptoms. However, the infant’s neurological status progressively deteriorated, and he began experiencing generalized tonic-clonic seizures. A contrast-enhanced brain CT scan revealed triventricular hydrocephalus, likely secondary to ventriculitis (Figure 1), and subsequent magnetic MRI confirmed the diagnosis of pyogenic ventriculitis (Figure 2).

The laboratory findings, which include elevated white blood cell, c-reactive protein (CRP) and procalcitonin levels, as well as renal function parameters and blood electrolytes, are summarized in the table below (Table 1). Urine testing was performed, including a urine culture (ECBU), which returned negative.

**Table 1:** Laboratory parameters compared to age-specific reference ranges (8-month-old infant).

Parameter	Measured Value	Reference Range (8-month-old infant)
Leukocytes (WBC)	31.500/mm³	6,000–17,500/mm³
C-Reactive Protein (CRP)	185 mg/L	<10 mg/L
Procalcitonin (PCT)	85.47 ng/L	<0.5 ng/L
Hemoglobin (Hb)	11.8 g/dL	11–13 g/dL
Sodium (Na)	130 mmol/L	135–145 mmol/L
Potassium (K)	3.6 mmol/L	3.5–5.6 mmol/L
Calcium (Ca)	2.23 mmol/L	2.1–2.6 mmol/L
Urea (Urea)	0.08 g/L	0.1–0.4 g/L
Creatinine (Creatinine)	24.31 µmol/L	20–40 µmol/L

Cerebrospinal fluid (CSF) analysis revealed a white blood cell count of 56,000/mm³ with neutrophilic predominance, hypoglycorrhachia (glucose level of 0.05 g/L), and elevated protein concentration (5.65 g/L). Bacteriological analysis of the cerebrospinal fluid, along with two blood cultures, revealed numerous colonies of multidrug-resistant *Escherichia coli*, confirming the diagnosis of *Escherichia coli* ventriculitis.

The infant was promptly intubated, placed on mechanical ventilation, and maintained under continuous sedation due to deterioration in both neurological and respiratory status. Valproic acid (Depakine) was initiated to manage seizure activity, and empirical broad-spectrum antibiotic therapy targeting nosocomial pathogens was commenced, consisting of amikacin (five days) and imipenem (15 days), supplemented with a short course of corticosteroids (dexamethasone). Given the multidrug-resistant *Escherichia coli* strain isolated from the CSF, which showed significant resistance to ceftriaxone, commonly used for central nervous system infections, the therapy was adjusted after clinical improvement and microbiological susceptibility results, with ciprofloxacin introduced as the follow-up treatment to complete the six weeks of antibiotic therapy.

To address the hydrocephalus and evacuate the purulent ventricular contents, an external ventricular drain was placed in the operating room. In addition to systemic antibiotic therapy, the child received intraventricular amikacin for three days via the external ventricular drain. A follow-up CT scan of the brain demonstrated regression of the hydrocephalus, along with evidence of transependymal resorption. The external ventricular drain (EVD) was removed after 12 days, following clinical improvement and resolution of the infection, as confirmed by negative cerebrospinal fluid cultures. After neurological improvement and successful extubation, the infant was transferred to the neurosurgery department for continued management with intravenous antibiotics and ongoing antiepileptic treatment.

## Discussion

Pyogenic ventriculitis is a bacterial infection of the cerebral ventricles, most commonly associated with head trauma, neurosurgical interventions, or the use of neurosurgical devices [3]. Although relatively rare, it may also arise as a complication of meningitis or brain abscess in pediatric patients. While neonatal ventriculitis is well documented in the literature, reports involving infants and older children, particularly those caused by *Escherichia coli*, remain scarce.

Primary pyogenic ventriculitis is infrequently encountered and has been linked to a range of pathogens, including *Neisseria meningitidis*, *Staphylococcus aureus*, and *Escherichia coli* [2]. A thorough literature review yielded no previously reported cases similar to ours, where an infant initially diagnosed with intussusception developed *Escherichia coli* ventriculitis secondary to postoperative sepsis.

CSF produced by the choroid plexus circulates through the subarachnoid spaces and is reabsorbed into the venous system. Hydrocephalus results when this flow is obstructed, which can occur at various anatomical levels; although rare, it may also result from CSF hypersecretion [4]. Inflammation of the meninges or ventricles due to infection or hemorrhage can impair CSF circulation and absorption, leading to hydrocephalus. Ventriculitis, in particular, can cause ependymal scarring, intraventricular obstruction, and the formation of multiloculated hydrocephalus [4,5].

*Escherichia coli*, a common cause of urinary tract infections and sepsis, is increasingly implicated in pediatric meningitis and may lead to pyogenic ventriculitis as a complication [6]. *Escherichia coli* translocation from the gastrointestinal tract to the CNS can occur via bacteremia, especially following abdominal surgery and compromised intestinal integrity. Once in the bloodstream, *Escherichia coli* can cross the blood-brain barrier through adhesion factors like OmpA and the K1 capsule, leading to CNS infections such as pyogenic ventriculitis. Biofilm formation and immune evasion further facilitate the development of these infections, even in the absence of direct brain injury [6].

Contrast MRI plays a pivotal role in diagnosing pyogenic ventriculitis. In contrast, CT scans are often normal or may reveal ventricular collections that are difficult to distinguish from blood or pus [3]. The MRI finding is purulent debris with irregular margins within the ventricles, appearing hyperintense on T1-weighted images, hypointense on T2-weighted images, and most clearly visualized on diffusion-weighted imaging (DWI) [7].

Given the wide range of potential bacterial pathogens, treatment regimens vary considerably. In some cases, surgical drainage of the ventricles is necessary [3]. The rarity of reported cases, diversity of causative organisms, and lack of standardized therapeutic guidelines make establishing a unified treatment protocol particularly challenging. However, an emerging consensus advocates for prolonged antibiotic therapy, typically lasting six to 12 weeks, similar to recommendations for brain abscesses as a critical component in the effective management of bacterial ventriculitis [6,8]. In cases where systemic antibiotics fail to adequately control the infection, particularly in the presence of loculated pus, intraventricular administration of antibiotics may serve as a valuable adjunct to ensure sufficient drug concentrations within the ventricular system [2].

## Conclusions

Pyogenic ventriculitis remains a diagnostic challenge due to its rarity, variable clinical presentation, and the wide spectrum of potential causative microorganisms. While it is most often associated with neurosurgical procedures or trauma, this case highlights a rare postoperative occurrence following abdominal surgery in an otherwise healthy infant.

MRI plays a crucial role in the early and accurate diagnosis of ventriculitis, particularly when CT findings are inconclusive or when clinical deterioration, such as evolving hydrocephalus or unexplained neurological symptoms, raises suspicion for central nervous system infection.

*Escherichia coli* ventriculitis following abdominal surgery is exceptionally rare and often underrecognized. In such cases, identifying potential sources of bacteremia, such as gastrointestinal translocation, is crucial. Management requires prolonged and targeted antibiotic therapy, and neurosurgical drainage combined with intraventricular antibiotics when indicated constitutes an essential component of care.
